# Uptake of long acting reversible contraception following integrated couples HIV and fertility goal-based family planning counselling in Catholic and non-Catholic, urban and rural government health centers in Kigali, Rwanda

**DOI:** 10.1186/s12978-020-00981-0

**Published:** 2020-08-17

**Authors:** Jeannine Mukamuyango, Rosine Ingabire, Rachel Parker, Julien Nyombayire, Andrew Abaasa, Gershim Asiki, Sarah Rae Easter, Kristin M. Wall, Laetitia Nyirazinyoye, Amanda Tichacek, Nadine Kaslow, Matt A. Price, Susan Allen, Etienne Karita

**Affiliations:** 1 Projet San Francisco, Rwanda Zambia HIV Research Group, Kigali, Rwanda; 2 Rwanda Zambia HIV Research Group, Department of Pathology & Laboratory Medicine, School of Medicine, Emory University, Atlanta, GA USA; 3 London School of Hygiene & Tropical Medicine (LSHTM), Keppel Street, London, WC1E 7HT UK; 4 Medical Research Council, Uganda Vaccine Research Institute & LSHTM Uganda Research Unit, Entebbe, Uganda; 5 Department of Obstetrics, Gynecology and Reproductive Biology, Brigham and Women’s Hospital, Harvard Medical School, Boston, MA USA; 6 Department of Epidemiology, Rollins School of Public Health, Emory University, Atlanta, GA USA; 7 University of Rwanda School of Public Health, Kigali, Rwanda; 8 Department of Psychiatry and Behavioral Sciences, School of Medicine, Emory University, Atlanta, GA USA; 9 International AIDS Vaccine Initiative, New York, NY USA; 10 Department of Epidemiology and Biostatistics, University of California at San Francisco, San Francisco, CA USA

## Abstract

**Background:**

When integrated with couples’ voluntary HIV counselling and testing (CVCT), family planning including long acting reversible contraceptives (LARC) addresses prongs one and two of prevention of mother-to-child transmission (PMTCT).

**Methods:**

In this observational study, we enrolled equal numbers of HIV concordant and discordant couples in four rural and four urban clinics, with two Catholic and two non-Catholic clinics in each area. Eligible couples were fertile, not already using a LARC method, and wished to limit or delay fertility for at least 2 years. We provided CVCT and fertility goal-based family planning counselling with the offer of LARC and conducted multivariate analysis of clinic, couple, and individual predictors of LARC uptake.

**Results:**

Of 1290 couples enrolled, 960 (74%) selected LARC: Jadelle 5-year implant (37%), Implanon 3-year implant (26%), or copper intrauterine device (IUD) (11%). Uptake was higher in non-Catholic clinics (85% vs. 63% in Catholic clinics, *p* < 0.0001), in urban clinics (82% vs. 67% in rural clinics, *p* < 0.0001), and in HIV concordant couples (79% vs. 70% of discordant couples, *p* = .0005). Religion of the couple was unrelated to clinic religious affiliation, and uptake was highest among Catholics (80%) and lowest among Protestants (70%) who were predominantly Pentecostal. In multivariable analysis, urban location and non-Catholic clinic affiliation, Catholic religion of woman or couple, younger age of men, lower educational level of both partners, non-use of condoms or injectable contraception at enrollment, prior discussion of LARC by the couple, and women not having concerns about negative side effects of implant were associated with LARC uptake.

**Conclusions:**

Fertility goal-based LARC recommendations combined with couples’ HIV counselling and testing resulted in a high uptake of LARC methods, even among discordant couples using condoms for HIV prevention, in Catholic clinics, and in rural populations. This model successfully integrates prevention of HIV and unplanned pregnancy.

## Plain English summary

Preventing unplanned pregnancy is an important health and economic goal for Africa. Contraceptive methods that don’t require remembering to take a pill every day or schedule an injection every two to 3 months are less likely to fail. Two of these methods, the copper intra-uterine device (IUD) and the hormonal implant, are effective for 12 years and 5 years, respectively. They can also be removed if a pregnancy is desired. Many African women could benefit from access to these long acting reversible contraceptive (LARC) methods but they require trained nurses to insert and male partners are usually not familiar with them. In government clinics in Kigali, the capital of Rwanda, couples who expressed a desire to wait at least 2 years before becoming pregnant were educated together about LARC methods first in a group and then individually with a nurse trained to insert LARC methods. The impact was substantial with 74% of 1290 couples requesting a LARC method. LARC methods are effective and affordable and programs that expand access are critically needed. Testing couples together for HIV is also a highly effective HIV prevention strategy and Rwanda is the only country in Africa that has nationalized this service in all government clinics. By combining: Couples’ voluntary HIV counseling and testing (CVCT) with Couples’ family planning counseling (CFPC), those that have one HIV-positive (HIV+) and one HIV-negative (HIV-) partner can be advised to use condoms for HIV prevention plus a LARC method for more effective pregnancy prevention.

## Background

Rwanda has the highest population density in continental Africa and a high burden of HIV, highlighting the need to mutually leverage HIV and family planning (FP) efforts. The modern contraceptive prevalence in married Rwandan women is estimated at 53.2% in 2015, with oral contraceptive pills (OCP) and progesterone-based injectables (usually depo-medroxyprogesterone acetate: DMPA or Depo-Provera) reported by most users (World Bank: https://data.worldbank.org/indicator/). With proactive social marketing and community-based distribution of OCP and DMPA, World Bank estimates show TFR has decreased from 8.5 in 1980 to 3.9 in 2015. To safeguard economic development, the Government of Rwanda (GoR) has set a goal of three or less for the total fertility rate (TFR).

For women who wish to end childbearing or delay pregnancy for more than 2 years, the most effective long-acting reversible contraceptives (LARC) available in Rwanda– the copper intrauterine device (IUD) and the hormonal contraceptive implant – are not subject to problems of user error or re-supply, and they are more effective and cost-efficient over the long term [1]. Once inserted by a clinician, the copper IUD is approved for up to 12 years of use. Contraceptive implants provide effective contraceptive coverage for 3 (Implanon) to 5 years (Jadelle) [2–5]. Though the majority of Rwandan couples want to limit or delay childbearing [6], few nurses are trained to insert LARC and as a result relatively few clients know about these methods [7, 8]. Lastly, many government health centers in Rwanda are affiliated with the Catholic Church and do not offer contraception.

The literature on HIV and FP integration includes interventions targeting providers [9–12], HIV+ patients [13–15], and discordant couples [16, 17] as well as efforts to enhance male involvement [18–20]. In heterosexual populations with a high prevalence of HIV, an important target audience for HIV and FP services is couples who can benefit from joint services [21–23]. Centers that provide integrated services report that clients vastly prefer this model [24, 25]. Integration models have added FP counseling and methods for patients in HIV care clinics [26–30] and conversely provided HIV testing and referral to FP clients [31–33]. However, historical, philosophical and structural differences in the fields of FP and HIV pose obstacles to integration both in the United States and developing countries [34]. Staff tend to see the two categories as distinctly different [35]. Service delivery styles also differ: FP clinics often rely on a fact-giving approach, while HIV-testing services emphasize client-centered counseling [36]. ‘Dual method’ use – condoms for HIV/STI prevention PLUS a longer-acting method for pregnancy prevention – is the best course of action [34], but often condom use is emphasized as an alternative rather than adjunctive method. Lastly, at the facility level, numerous studies confirm that logistical obstacles including under-staffing, lack of space, vertical service silos and sub-optimal training remain major challenges [37–45].

Our previous work has shown that LARC training and promotion in Kigali government clinics increased the proportion of family planning clients who requested a LARC method from < 2% in 2009 to 17% in 2014 [46], confirming that LARC are acceptable to women seeking contraception. We have also shown that limited knowledge of LARC methods among men [7] and poor communication about fertility goals within the couple [47] can be overcome through joint counseling, leading to LARC uptake [6]. While these advances are promising, women and couples at risk of unplanned pregnancy in Rwanda continue to face limited information and access, particularly in rural areas and in clinics with Catholic affiliation [48]. The impact of this is reflected in the (illegal) abortion rate of 25/1000 women annually [49].

Similar to other African countries [50–53], there is consensus among stakeholders in Rwanda that HIV and family planning (FP) services should be integrated [54]. The Government of Rwanda strongly supports access to the full range of contraceptive options [55] and provides free therapeutic antiretrovirals (ARV) and PMTCT for HIV [56]. Rwanda provides a unique context in which to evaluate HIV-FP integration: since 2013, two-thirds of new HIV infections in marriage have been prevented by nationalized Couples Voluntary HIV Counseling and Testing (CVCT) in antenatal clinics [57–59]. This addresses prong 1 of prevention of mother-to-child transmission (PMTCT) – (prevention of HIV in childbearing women). Prong 2 of PMTCT focuses on prevention of unplanned pregnancies among couples with or at risk of HIV. Integrated services that emphasize effective contraception and dual method use would maximize both Prongs 1 & 2 of PMTCT [60] and cost less per HIV infection averted than Prongs 3 and 4 that involve ARVs [61–64]. However, in Rwanda, CVCT is not currently offered to non-pregnant couples, and contraception is provided to women in family planning clinics but not elsewhere.

We present here the results of an integrated program providing 1) joint HIV testing and family planning counseling; 2) fertility-goal based LARC promotion and provision and 3) training, supplies and protected time for service providers. This program focuses on both supply and demand addressed simultaneously with skills training for providers, education for clients, and adequate human resources and space. To explore potential obstacles and facilitators, we compare LARC uptake in relation to rural/urban clinic location, Catholic/non-Catholic clinic affiliation, couple HIV serostatus and other participant characteristics.

## Methods

### Study design and setting

This observational study of the effect of an intervention on LARC uptake [65] was conducted by the Projet San Francisco (PSF), a part of the Rwanda Zambia HIV Research Group (RZHRG) established in 1986 and affiliated with the Emory University School of Medicine, in collaboration with the Ministry of Health (MoH) of Rwanda. We selected eight health centers in and around the city of Kigali offering CVCT in antenatal clinics (ANC), infant vaccination, quarterly follow-up of HIV sero-discordant couples, and HIV care and antiretroviral treatment (ART) services. We chose two health centers in each of the following categories, ensuring that no catchment areas overlapped: urban Catholic, urban non-Catholic, rural Catholic, and rural non-Catholic. Catholic-affiliated clinics do not provide family planning but refer to nearby health posts established by the MoH for that purpose. Clinic nurses screened couples in outpatient departments and referred eligible and interested couples to the study. All activities and data collection took place at the government clinics.

To ensure that clinic staff providing HIV and/or FP services were not distracted from other responsibilities, we hired them to work for the study while they were off duty. Training in counseling and LARC insertion and removal was provided with certificates for successful trainees. This protected staff from overburden and patients from unnecessary delays. Clinic stocks of LARC supplies including insertion kits and autoclaves were assessed and necessary provisions provided as needed.

### Study population

We enrolled heterosexual cohabiting couples in which the woman was aged between 21 and 40 years and the man aged > = 21; planning to live in Kigali for at least 2 years; both partners were fertile (prior history of pregnancy, no surgical sterilization or hysterectomy); the woman was not pregnant; the couple was not wanting to conceive in the next 2 years; and the woman was not using a LARC method. Our enrollment was targeted to reach 1200 couples equally distributed between concordant HIV positive (M + F+), concordant HIV- (M-F-), and discordant (M + F- and M-F+) between May and December of 2015.

### Study procedures

On the day of enrollment, study counselors conducted group information sessions to read and explain the informed consent document to interested couples. Couples then discussed with a counselor privately and signed a joint informed consent. Interviewers administered a baseline questionnaire to the man and the woman separately to collect data on demographic characteristics, obstetric and contraceptive history, fertility goals and knowledge and beliefs about LARC methods. Interviewers used tablet-based data collection with survey CTO. Couples were tested for HIV (two rapid tests according to the national algorithm) [66] and syphilis (RPR, Macro-vue) [67].

### Intervention

While couples were waiting for their serology test results, a group session was conducted by a trained community health worker (CHW) using a flip chart. The MoH had previously trained Kigali CHW to distribute OCP and administer injectable contraceptives in their communities. Study nurses provided them with additional LARC information prior to study initiation [68]. The flip chart presented a fertility goal-based approach to contraceptive choice, with the advantages of LARC methods highlighted for this audience of couples wishing to limit or delay pregnancy for at least 2 years. The flip chart illustrated IUD and implant insertion procedures and discussed contraindications and side effects. Jadelle and Implanon Implants and copper IUD (LARC methods available through government procurement) were passed around the group and participants were encouraged to feel them and to ask questions.

Following the group session, nurse-counselors provided HIV and syphilis post-test counseling to both partners together with appropriate treatment and referral. They then asked a series of questions structured to help the couple discuss and agree on fertility goals and decide whether a LARC method would be suitable for them. Couples could choose to have a LARC method inserted immediately or could request a subsequent appointment for insertion.

### Measurements

The main outcome of the study was uptake of any of the LARC (Jadelle, Implanon or IUD) methods on the day of enrollment or any time prior to the follow-up visit scheduled 1 month later. The study primary exposures were rural/urban clinic location, Catholic/non-Catholic clinic affiliation, and couple HIV serostatus. Demographic measures assessed on baseline questionnaire included age, years of cohabitation, number of children, duration of residence in Kigali, literacy in Kinyarwanda, English and French, level of education, income, religious affiliation, and frequency of church attendance. Prior HIV testing history, ART use, contraceptive use and source of information, fertility goals and knowledge, and previous experience or discussion about LARC methods were recorded.

### Statistical analysis

Statistical analysis was performed in SAS v9.4 (Cary, NC). The association between the outcome (uptake of LARC) and categorical variables was determined using chi-square or Fisher’s exact for categorical variables, and two-tailed t-test was used for continuous variables. Frequencies of responses are shown in Tables as column percent. LARC uptake within response categories are shown in Tables as row percent. Bivariate and multivariate logistic regression models were used to estimate crude and adjusted odds ratios, 95% confidence intervals (CIs), and *p*-values. An interaction term was included for clinic location and clinic religion based on differences in association with LARC uptake among sub-groups. Covariates significant in bivariate models at an alpha of 0.05 were included in the multivariate model following a collinearity assessment; covariates not significant in the multivariate model at an alpha of 0.05 were removed via backwards elimination from the final multivariate model.

### Ethical consideration

Ethical approval was obtained from the National Ethics Committee of Rwanda and the Institutional Review Board at Emory University. All participants signed the approved, informed consent document before enrollment. Participant data was unlinked to identifiers and kept confidential.

## Results

Unless specified, all comparisons mentioned are statistically significant with *p*-values shown in Tables, footnotes or text. Variables not significantly associated with LARC uptake are listed in Table footnotes.

### LARC uptake by couple HIV serostatus, urban vs. rural clinic location, and Catholic vs. non-Catholic clinic affiliation (Table 1)

Of 1691 couples referred from infant vaccination (54%), follow-up of discordant couples (35%), HIV care and treatment (9%) and family planning (2%) services, 1353 (80%) were screened and 1290 (76%) were eligible and enrolled.

**Table 1:** LARC uptake by couple serostatus, urban vs.rural location and Catholic vs.non-Catholic clinic affiliation

	All Couples (*n* = 1290)		LARC uptake at enrollment (*n* = 767)		LARC uptake within median 30 days (*n* = 193)		*p*-value*: LARC uptake at enrollment vs. within 30 days	No LARC uptake (*n* = 330)		*p*-value*: Across all sub-groups
	n	Column %	n	Row %	n	Row %		n	Row %	
**Couple HIV status**							0.2049			0.0005
M-F-	330	26%	218	66%	46	14%		66	20%	
M + F+	327	25%	198	61%	54	17%		75	23%	
M-F+	319	25%	181	57%	57	18%		81	25%	
M + F-	314	24%	170	54%	36	11%		108	34%	
**Clinic affiliation and location**										
Urban							< 0.0001			< 0.0001
Catholic	315	24%	148	47%	66	21%		101	32%	
Non-Catholic	333	26%	284	85%	31	9%		18	5%	
Rural							0.6749			< 0.0001
Catholic	317	25%	142	45%	43	14%		132	42%	
Non-Catholic	325	25%	193	59%	53	16%		79	24%	

*Chi-square test for categorical variables

These included 330 (26%) HIV concordant negative (M-F-), 327 (25%) HIV concordant positive (M + F+), 319 (25%) (woman positive partner; M-F+), and 314 (24%) HIV discordant (man positive partner; M + F-). Each serotatus was equally distributed among urban Catholic (*n* = 315, 24%), urban non-Catholic (*n* = 333, 26%), rural Catholic (*n* = 317, 25%) and rural non-Catholic (*n* = 325, 25%) clinics.

LARC uptake was higher among concordant (79%) than among discordant couples (70%) (*p* = 0.0005), and among couples with HIV- men (77%) compared with HIV+ men (71%) (*p* = 0.0152). Concordant HIV- couples were the most likely to uptake LARC methods on the day of enrollment (66% vs 57% of other couples, *p* = 0.0046). Compared to all other couples, M + F- couples were significantly less likely to uptake LARC (66% vs. 77% of other couples, *p* = < 0.0001).

Uptake was higher in non-Catholic clinics (85% vs 63% in Catholic clinics, *p* < 0.0001), and in urban clinics (82% vs 67% in rural clinics, *p* < 0.0001). LARC uptake was highest among urban non-Catholic clinics (95%), followed by rural non-Catholic clinics (76%), urban Catholic clinics (68%), and the lowest uptake was 58% in rural Catholic clinics (*p* < 0.0001). As expected, uptake in urban Catholic clinics was more likely to be after the date of enrollment (21% vs 9% in urban non-Catholic clinics, *p* < 0.0001) as it required a referral to the nearby health post which could not always be acted on the same day. Uptake at enrollment was the same in urban (47%) and rural (45%) Catholic clinics but lower after enrollment in rural areas (14% vs 21% in urban Catholic clinics, *p* = 0.014). This may reflect the greater obstacle presented by distance in rural areas.

### Demographic factors associated with LARC uptake after the intervention (Table 2)

Mean ages were 31 for women and 35–36 for men, with concordant negative couples having the youngest mean ages compared to all other serostatus groups (29.3 vs. 31.0, *p* < 0.0001 and 33.3 vs. 37.3, *p* < 0.0001, for women and men respectively). Mean duration of union was 6–7 years with concordant positive couples having the longest mean duration of cohabitation compared to all other serostatus groups (7.8 vs. 6.8, *p* = 0.0020) and concordant negative couples the shortest mean duration compared to all other serostatus groups (6.3 vs. 7.3, *p* = 0.0009). The mean number of biological children was 2.2 with no significant differences between serostatus groups.

**Table 2:** Significant individual and couple-level demographic factors associated with LARC uptake after the intervention

	All Couples (*n* = 1290)		LARC uptake at baseline or within median 30 days (*n* = 960)		Did not uptake LARC (*n* = 330)		*p*-value*
	mean	SD	mean	SD	mean	SD	
**Man’s Age**	36.3	8.2	35.7	8.2	37.9	8.2	< 0.0001
**Woman’s Age**	30.6	5.4	30.4	5.5	31.1	5.1	0.0240
**Man’s Years in Kigali**	16.9	11.8	16.1	11.6	19.1	11.9	< 0.0001
**Woman’s Years in Kigali**	13.8	10.1	13.3	10.1	15.3	10.2	0.0017
**Cohabitation (years)**	7.1	5.0	6.7	4.9	8.2	5.1	< 0.0001
**Number of biological children**	2.2	1.4	2.2	1.3	2.4	1.5	0.0190
**Number of children in household**	2.8	1.6	2.7	1.5	3.0	1.6	0.0004
	n	Column %	n	Row %	n	Row %	
**Man Reads Kinyarwanda**							0.0144
Easily/with difficulty	1005	78%	732	73%	273	27%	
Not at all	285	22%	228	80%	57	20%	
**Man Reads French**							0.0066
Easily/with difficulty	198	15%	132	67%	66	33%	
Not at all	1092	85%	828	76%	264	24%	
**Man’s Level of Education**							0.0004
Primary/Secondary/Tertiary	813	63%	578	71%	235	29%	
No education	477	37%	382	80%	95	20%	
**Woman’s Level of Education**							0.0225
Primary/Secondary/Tertiary	752	58%	542	72%	210	28%	
No education	538	42%	418	78%	120	22%	
**Man’s Religion**							0.0005
Catholic/Other	635	49%	500	79%	135	21%	
Pentecostal/Seven Day Adventist/Jehovah’s Witnesses/Anglican/Baptist/Muslim/No religion	655	51%	460	70%	195	30%	
**Woman’s Religion**							0.0019
Catholic/Other	535	41%	432	81%	103	19%	
Pentecostal/Seven Day Adventist/Jehovah’s Witnesses/Anglican/Baptist/Muslim/No religion	755	59%	528	70%	227	30%	
**Couple Religion**							< 0.0001
Both Catholic/Other	425	33%	340	80%	85	20%	
Woman Catholic/Other, Man Pentecostal/Seven Day Adventist/Jehovah’s Witnesses/Anglican/Baptist/Muslim/No religion	110	9%	92	84%	18	16%	
Man Catholic/Other, Woman Pentecostal/Seven Day Adventist/Jehovah’s Witnesses/Anglican/Baptist/Muslim/No religion	210	16%	160	76%	50	24%	
Both Pentecostal/Seven Day Adventist/Jehovah’s Witnesses/Anglican/Baptist/Muslim/No religion	545	42%	368	68%	177	32%	
**Man frequency of attending religious services**							0.0001
More than once a week	272	21%	178	65%	94	35%	
Once a week or less often	1018	79%	782	77%	236	23%	
**Woman frequency of attending religious services**							< 0.0001
More than once a week	392	30%	262	67%	130	33%	
Once a week or less often	898	70%	698	78%	200	22%	
**Man’s frequency of attending religious services by religion**							
Catholic/Other							0.0578
More than once a week	66	10%	46	70%	20	30%	
Once a week or less often	569	90%	454	80%	115	20%	
Pentecostal/Seven Day Adventist/Jehovah’s Witnesses/Anglican/Baptist/Muslim/No religion							0.0197
More than once a week	206	31%	132	64%	74	36%	
Once a week or less often	449	69%	328	73%	121	27%	
**Woman’s frequency of attending religious services by religion**							
Catholic/Other							0.3745
More than once a week	98	18%	76	78%	22	22%	
Once a week or less often	437	82%	356	81%	81	19%	
Pentecostal/Seven Day Adventist/Jehovah’s Witnesses/Anglican/Baptist/Muslim/No religion							0.0014
More than once a week	294	39%	186	63%	108	37%	
Once a week or less often	461	61%	342	74%	119	26%	

*Two-tailed t-test for continuous variables and chi-square test for categorical variables

The following demographic covariates were analyzed but were not associated with LARC uptake in chi-square analysis and are not presented above: man has income; woman has income, woman’s literacy in English, French, and Kinyarwanda; man’s literacy in English

Couples who requested LARC were on average younger (35.7 vs. 37.9 for men, 30.4 vs. 31.1 for women), had lived in Kigali (16.1 vs.19.1 years for men, 13.3 vs.15.3 for women), and cohabited for fewer years (6.7 vs. 8.2 years) and had fewer children (2.2 biological and 2.7 in the home vs. 2.4 biological and 3.0 in the home for non-LARC couples). These differences were all statistically significant.

Not completing primary school was reported by 42% of women and 37% of men, with concordant HIV- couples having the highest educational achievements and concordant positive couples the lowest (*p* < 0.0001 for both men and women). Literacy results were similar with 66% of men and 61% of women able to read easily in Kinyarwanda but a substantial difference between M-F- couples (80% of men and 76% of women read easily) and M + F+ couples (54% of men and 48% of women) (*p* < 0.0001 for both men and women). Discordant couples had intermediate literacy with 58–68% reporting easily reading Kinyarwanda. Reading easily in English or French was uncommon (2–4% overall) with M-F- couples again having the highest literacy (7–8%) and M + F+ couples the lowest (1–2%) (*p* < 0.0001 for both men and women). Most (94%) men and 43% of women reported some income with a monthly median of 40,000 Rwandan francs ($50) for men and 20,000 Rwandan francs ($25) for women (not shown).

Table 2 presents significant individual and couple-level demographic factors associated with LARC uptake after the intervention. Variables not associated with LARC appear as footnotes to the table. Among both men and women, LARC uptake was highest in those with no education (80% of men and 78% of women) compared with those who had completed at minimum primary school (71% of men and 72% of women). Among men, literacy in Kinyarwanda and French was associated with lower LARC uptake (73% vs. 80% of illiterate and 67% vs. 76% of illiterate, respectively).

The most commonly reported religious affiliation among men was Catholicism (44% vs. 34% of women, *p* < 0.0001), while women were more likely to report being Pentecostal (36% vs. 23% of men, *p* < 0.0001). There were no notable differences in religious affiliation between couple HIV status groups. Other Protestant denominations included 9% Seventh Day Adventist, 4% Anglican, 1% each Jehovah witnesses and Baptist, with no difference between genders. Fifteen percent of participants were not Catholic nor Protestant: this included Muslims (6%), other (6%) and those reporting no religious affiliation (3%). LARC uptake was similar in all Protestant groups and in the Muslim/other/no religion categories and these are combined in Table 2.

Religion and frequency of attending religious services were associated with LARC uptake among both men and women, with Catholics (78% of men and 80% of women) having the highest and Protestants (70% of men and 71% of women) the lowest LARC uptake. Attending services more than once a week was also associated with lower LARC uptake (65–67% of men and women, respectively) compared with those attending services once a week or less (77% of men and 78% of women). Among men this trend was noted in all religious groups though with only borderline significance among Catholics. In contrast, among women, only Protestants who attended services more than once/week had lower LARC uptake than those attending only once per week or less.

### HIV status, fertility goals, contraceptive experiences and concerns associated with LARC uptake after the intervention (Table 3)

As reported above, couples with HIV+ men were less likely to uptake LARC. Among HIV+ men reporting ARV use, LARC uptake was lower (69% vs. 85% of couples with non-ARV-using HIV+ men). These associations with HIV status and ARV use were not noted for women.

**Table 3:** HIV status, fertility goals, contraceptive experiences and concerns associated with LARC uptake after the intervention

	All Couples (*n* = 1290)		LARC uptake at baseline or within median 30 days (*n* = 960)		Did not uptake LARC (*n* = 330)		*p*-value*
	n	Column %	n	Row %	n	Row %	
**Man’s HIV Status**							0.0152
Positive	641	50%	458	71%	183	29%	
Negative	649	50%	502	77%	147	23%	
**Of HIV+, man on ARV**							0.0001
Yes	537	84%	370	69%	167	31%	
No	104	16%	88	85%	16	15%	
**Plan to have more children**							0.0283
Yes/Unsure	634	49%	489	77%	145	23%	
No	656	51%	471	72%	185	28%	
**Previously discussed family planning as a couple**							< 0.0001
Yes	1201	93%	913	76%	288	24%	
No	89	7%	47	53%	42	47%	
**Couple previously discussed**							
IUD	129	10%	116	90%	13	10%	< 0.0001
Jadelle	548	42%	517	94%	31	6%	< 0.0001
IUD or Jadelle	628	49%	589	94%	39	6%	< 0.0001
**Man: Sources of information about contraceptive methods**							
Community Health Worker	398	31%	310	78%	88	22%	0.0563
Radio	406	31%	281	69%	125	31%	0.0037
Newspaper	36	3%	19	53%	17	47%	0.0025
**Current method to prevent pregnancy**							0.0001
Injectable	322	25%	221	69%	101	31%	
Condoms	591	46%	427	72%	164	28%	
OCP	93	7%	76	82%	17	18%	
Other/None	284	22%	236	83%	48	17%	
**Man: Concerns about implant**							0.0038
Negative side effects	188	16%	124	66%	64	34%	
Bad for health	70	6%	51	73%	19	27%	
Doesn’t work	39	3%	33	85%	6	15%	
Other	14	1%	6	43%	8	57%	
No concerns	653	55%	497	76%	156	24%	
Don’t know	229	19%	172	75%	57	25%	
**Woman: Concerns about implant**							< 0.0001
Negative side effects	292	23%	184	63%	108	37%	
Bad for health	93	7%	70	75%	23	25%	
Doesn’t work	59	5%	44	75%	15	25%	
Other	15	1%	9	60%	6	40%	
No concerns	638	50%	506	79%	132	21%	
Don’t know	171	13%	132	77%	39	23%	
**Man: Concerns about IUD**							0.0031
Negative side effects	74	11%	54	73%	20	27%	
Bad for health	49	7%	45	92%	4	8%	
Doesn’t work	45	6%	42	93%	3	7%	
Other	12	2%	8	67%	4	33%	
No concerns	317	45%	228	72%	89	28%	
Don’t know	200	29%	151	76%	49	25%	
**Woman: Concerns about IUD**							0.0665
Negative side effects	166	15%	109	66%	57	34%	
Bad for health	132	12%	98	74%	34	26%	
Doesn’t work	102	9%	80	78%	22	22%	
Other	39	4%	29	74%	10	26%	
No concerns	419	38%	309	74%	110	26%	
Don’t know	235	22%	186	79%	49	21%	

*Two-tailed t-test for continuous variables and chi-square test for categorical variables

The following covariates were analyzed but were not associated with LARC uptake in chi-square analysis and are not presented above: woman’s HIV or ARV status; individual or couple RPR status; preferred timing of last pregnancy; man wants more children (yes/don’t know vs no); woman wants more children (yes/don’t know vs no); all women’s sources of information about contraceptive methods; clinic staff, friends, spouse, TV or other as men’s sources of information about contraceptive methods; man previously heard of implant; woman previously heard of implant; man previously heard of IUD; woman previously heard of IUD; woman previously used implant; and woman previously used IUD

Man and woman’s concerns about negative side effects from the implant and IUD were included in bivariate and multivariate models as dichotmous variables (concerned about negative side effects yes/no)

Somewhat counter-intuitively, planning to have more children was associated with higher LARC uptake (77% vs. 72% of those not planning to have more children). For men, the most common source of information about family planning was the clinic, CHW (associated with higher LARC uptake), or radio (associated with lower LARC uptake). Half of couples had previously discussed LARC together and this was associated with a 94% uptake of LARC methods compared with only 54% among couples who had not discussed IUD or implant previously. Current use of injectable contraception or condoms as the only contraception prior to the intervention was associated with lower uptake of LARC methods (69 and 72%, respectively compared with 82–83% of OCP and non-modern method users). Among the 92% of men and 98% of women who had previously heard of the implant, 16% of men and 23% of women thought they had negative side effects and this was associated with lower LARC uptake (66% of men and 63% of women compared with 76% of men and 78% of women not citing negative side effects as a concern, respectively).

### Multivariate analysis of factors associated with LARC uptake (Table 4)

Variables associated with LARC uptake in bivariate analysis were included in the multivariate model with the exception of man’s ARV use which was collinear with man’s HIV status. Interaction terms between rural/urban and Catholic/non-Catholic clinics showed non-Catholic clinics having higher uptake in both urban and rural areas and urban having higher uptake than rural in this group. In this model, the interaction term for urban vs. rural LARC uptake in Catholic clinics was not significant. Couple HIV status did not remain associated with LARC uptake in the multivariate model. Only the age of the man remained significant when both men’s and women’s ages and number of children were included in the model. Not having completed at least primary school remained predictive of LARC uptake among both men and women. When religious affiliation of the man and woman were combined, couples with the woman or both partners Catholic remained significantly more likely to choose a LARC method. Frequency of attending religious services did not remain independently predictive in the final multivariate model. Baseline injectable and condom use remained predictive of lower LARC uptake compared with OCP use or use of traditional or no contraception. The strongest predictor of LARC uptake was the couple having discussed one or both LARC methods prior to the day of enrollment. Higher LARC uptake was also associated with women not having concerns about implant negative side effects.

**Table 4:** Multivariate analysis of clinic, couple and individual-level factors associated with LARC uptake after the intervention

	Bivariate models				Final multivariate model			
		95% CI				95% CI		
	cOR^a^	LL^b^	UL^c^	*p*-value	aOR^d^	LL^b^	UL^c^	*p*-value
Clinic Location*Clinic Religion								
Non-Catholic vs Catholic in Urban Clinics	8.26	4.86	14.04	< 0.0001	4.16	2.34	7.39	< 0.0001
Non-Catholic vs Catholic in Rural Clinics	2.22	1.58	3.11	< 0.0001	1.47	1.01	2.16	0.0447
Urban vs Rural in Non-Catholic Clinics	5.62	3.28	9.63	< 0.0001	2.84	1.58	5.11	0.0005
Urban vs Rural in Catholic Clinics	1.51	1.09	2.09	0.0128	1.01	0.70	1.46	0.9666
**Couple HIV Status**								
M-F-	*ref*	–	–	–				
M + F+	0.84	0.58	1.22	0.3602				
M + F-	0.48	0.33	0.68	< 0.0001				
M-F+	0.74	0.51	1.06	0.1017				
**Man on ARV**								
Yes	1.63	1.27	2.10	0.0001				
No	*ref*	–	–	–				
**Man Age (per one year increase)**	0.97	0.96	0.98	< 0.0001	0.97	0.95	0.99	0.0011
**Woman Age (per one year increase)**	0.97	0.95	1.00	0.0242				
**Cohabitation (per one year increase)**	0.95	0.92	0.97	< 0.0001				
**Man’s Years in Kigali (per one year increase)**	0.98	0.97	0.99	< 0.0001				
**Woman’s Years in Kigali per one year increase)**	0.98	0.97	0.99	0.0018				
**Number of Biological Children (per each child increase)**	0.89	0.82	0.98	0.0123				
**Number of Children in Household (per each child increase)**	0.87	0.81	0.94	0.0005				
**Man’s Highest Education Completed**								
None	1.64	1.25	2.14	0.0004	1.39	1.01	1.90	0.0451
Primary/Secondary/Higher	*ref*	–	–	–				
**Woman’s Highest Education Completed**								
None	1.35	1.04	1.75	0.0228	1.53	1.13	2.08	0.0067
Primary/Secondary/Higher	*ref*	–	–	–				
**Man Reads Kinyarwanda**								
Easily/With Difficulty	*ref*	–	–	–				
Not at all	1.49	1.08	2.06	0.0149				
**Man Reads French**								
Easily/With Difficulty	*ref*	–	–	–				
Not at all	1.57	1.13	2.17	0.0069				
**Couple Religion**								
Both Catholic/Other	1.92	1.42	2.59	< 0.0001	1.53	1.09	2.16	0.0152
Woman Catholic/Other, Man Pentecostal/Seven Day Adventist/Jehovah’s Witnesses/Anglican/Baptist/Muslim/No religion	2.46	1.44	4.20	0.001	2.05	1.13	3.74	0.0190
Man Catholic/Other, Woman Pentecostal/Seven Day Adventist/Jehovah’s Witnesses/Anglican/Baptist/Muslim/No religion	1.54	1.07	2.22	0.0205	1.40	0.92	2.13	0.1212
Both Pentecostal/Seven Day Adventist/Jehovah’s Witnesses/Anglican/Baptist/Muslim/No religion	*ref*	–	–	–				
**Man frequency of attending religious services**								
Once a week or less often	1.75	1.31	2.34	0.0001				
More than once a week	*ref*	–	–	–				
**Woman frequency of attending religious services**								
Once a week or less often	1.73	1.33	2.52	< 0.0001				
More than once a week	*ref*	–	–	–				
**Plan to have more children together**								
Yes/unsure	1.33	1.03	1.7	0.0285				
No	*ref*	–	–	–				
**Contraceptive on day of enrollment**								
Injectable	0.45	0.3	0.66	< 0.0001	0.55	0.35	0.87	0.0096
Condoms	0.53	0.37	0.76	0.0005	0.59	0.39	0.89	0.0113
OCP	0.91	0.49	1.67	0.7601	1.21	0.61	2.40	0.5935
Other/None	*ref*	–	–	–				
**Couple previously discussed IUD and/or implant**								
Yes	11.85	8.28	16.95	< 0.0001	7.59	5.18	11.13	< 0.0001
No	*ref*	–	–	–				
**Man heard of implant and concerned about side effects**								
No	1.62	1.16	2.26	0.0042				
Yes	*ref*	–	–	–				
**Woman heard of implant and concerned about negative side effects**								
No	2.05	1.55	2.72	< 0.0001	1.49	1.07	2.07	0.0193
Yes	*ref*	–	–	–				
**Woman heard of IUD and concerned about negative side effects**								
No	1.63	1.15	2.31	0.0059				
Yes	*ref*	–	–	–				

The following variables were not significant in bivariate analyses and are not tabled: woman HIV+ or on ARV, man wants more children, woman wants more children, woman previously used IUD, woman previously used implant, preferred timing of last pregnancy, man concerned about negative side effects of IUD

^a^Crude odds ratio

^b^Lower limit for 95% confidence interval (CI)

^c^Upper limit for 95% confidence interval (CI)

^d^Adjusted odds ratio

## Discussion

Our intervention used a coordinated, multifaceted approach to HIV-FP integration building on the literature and prior experience in Rwanda. Key components were: including both cohabiting partners, triaging participants based on fertility goals, providing low cost group education by CHW, cross-training FP and HIV clinic staff in client-centered approaches and dual-method counseling for HIV discordant couples, training FP nurses to comfortably insert LARC methods, procurement of LARC supplies, point-of-care LARC insertion, and protected government clinic staff time. Our results confirm that when integrated with joint HIV counseling and testing, a simple intervention offering LARC to couples who desire to delay or limit pregnancy results in a high uptake of these methods. Overall uptake was encouraging ranging from 58% in rural Catholic clinics to 95% in urban, non-Catholic clinics. Residual obstacles at the facility- and client-level are addressable, as discussed below.

The literature on HIV-FP integration at the facility-level highlights the importance of structural aspects of service delivery including commodities and reagent stocks, provider knowledge and skills, workload and job satisfaction [42, 43]. In Tanzania significant determinants of facility readiness for integrating FP and HIV were being government owned, having routine management meetings, availability of guidelines in-service training of staff and availability of laboratories for HIV testing [37]. In Kenya, facilities where staff were supported by management to work as a team to share workload delivered better integrated services despite resource shortages. In contrast, where staff were poorly organized and unsupported, services were poor despite structural integration [40]. A South African group developed a model for KwaZulu-Natal that provided capacity building and commodity monitoring with involvement of community members [41], while a quality improvement program for maternal, child and HIV care in South African primary health care facilities resulted in improved child services but no impact on HIV-FP integration [39]. A comparison of FP services in integrated and non-integrated clinics in Malawi and Tanzania found no adverse outcomes due to strain on facility or providers but noted strengthened FP commodity stocks likely due to HIV-related supply chains in integrated clinics [38]. An organizational network analysis in Ethiopia [44] led to an intervention that resulted in a 55% increase in HIV-FP referrals [45].

These experiences make clear the critical role facility and staff components play in the success of any HIV-FP program. Our intervention included capacity building and ensured that the necessary commodities were present. Hiring successful trainees when they were off duty provided a financial incentive and allowed staff to become confident and proud of their newly acquired skills. While some would argue that this approach is not sustainable, we found during the nationwide expansion of CVCT that new skills transition into day to day practice and – when supported by MoH mandates – eventually become the standard of care [59]. We also implemented our integrated HIV-FP model in 55 urban and 215 rural government clinics across 33 districts in Zambia: the program provided HIV-FP counseling to 208,211 couples and performed 101,322 LARC insertions [69].

Though promising, our results do expose facility level obstacles to HIV-FP integration in Rwanda, specifically the influence of Catholic affiliation and rural location. Prior to the Genocide of the Tutsi in 1994, Rwanda was predominantly Catholic [70]. Since the Genocide, the demography of religion in Rwanda has changed with a marked increase in the number of Protestants denominations, in particular Pentecostal churches [71] which were particularly popular among women in our sample. Clinics affiliated with the Catholic Church do not provide contraception but they do refer interested clients to a nearby health post established by the MoH for family planning [72]. Staff are provided by the MoH without regard to their religion and rotated regularly, therefore we presume that provider bias did not operate differentially in Catholic and non-Catholic clinics. Interestingly, the proportion of Catholic clients did not differ in Catholic and non-Catholic clinics, and Catholic women and couples were more receptive to LARC than others as has been reported by others [73, 74]. The uptake of LARC among our study participants referred from Catholic clinics to health posts, though low relative to non-Catholic clinics, was still substantial. The lower uptake in Catholic clinics was thus most likely due to the need to travel to the nearby health post rather than to any effect of personal religious beliefs.

At the client-level, time and distance were likely contributors to lower uptake in rural compared with urban clinics. Shifting the task of LARC endorsement to CHW is one solution to distance and physically separated services. In a related study in 8 different catchment areas in Kigali, we used the same training procedures for CHW as was used here, but tasked them with educating the households they were assigned to about LARC and providing written referrals for interested women and couples. Specifically, the clients they provided OCP and DMPA injections to, along with women who were post-partum or multigravida, were prioritized as target audiences that might benefit from learning about LARC [68]. In a 13 month period, CHW distributed 7712 referrals, 79% leading to clinic visits and 95% of those resulting in LARC uptake. Of note, CHW could advise interested women to go directly to the health post (rather than the Catholic clinic), and could emphasize that while traveling for LARC insertion might be burdensome, one visit resulting in long acting protection was preferable to repeated visits or a method failure.

LARC-focused content of the education sessions, targeted messaging and the focus on couples rather than women alone would not have succeeded without the structural underpinning. That said, we did identify individual and couple-level characteristics of associated with LARC uptake and indicative of gaps that should be examined.

The strongest predictor of LARC uptake was having previously discussed LARC methods as a couple, which reinforces the need to expand demand creation strategies while bolstering clinic capacity to provide LARC methods. However, concern about the negative side effects of hormonal implants remained a deterrent to LARC uptake among women. Overcoming misconceptions and knowledge gaps among providers and patients is an important and ongoing need that should be addressed by CHW and clinic staff [75].

Concordant couples were more likely to uptake LARC than discordant couples, the latter being more likely to report condom use as their only contraceptive method. Couple HIV status was likely confounded with condom use and only the latter remained predictive in the multivariate analysis. Use of DMPA as contraception was also independently associated with not choosing LARC. It may be that condom and DMPA users were satisfied with their current method and did not feel the need to have additional protection against unplanned pregnancy. However, condoms have a higher contraceptive failure rate than other modern methods, even among discordant couples who are highly motivated to adhere with them consistently [76–78], and previous studies have reported more unprotected sex in DMPA vs non-hormonal method users [79]. More impactful dual-method counseling for condom and DMPA users may be an important improvement. Additionally, previous studies have noted high rates of discontinuation among DMPA users [80] and further study is needed to assess whether a LARC method might be an acceptable alternative to this subgroup.

The limitations of our study included limited generalizability of our findings given convenience sampling of couples expressing a desire to delay or limit pregnancy, limited sample size to explore some association, and loss to follow-up that may be differential by exposures of interest.

## Conclusions

A combination of couples-focused education about LARC based on stated fertility goals and access to point of care LARC insertion was highly impactful in Kigali, Rwanda. Combining this program with joint HIV testing and counseling mutually leverages heterosexual and perinatal HIV prevention and unplanned pregnancy prevention. We encourage further research, both behavioral and operational, to further optimize and integrate HIV and unplanned pregnancy prevention in resource-limited settings.

## Data Availability

The datasets used and/or analyzed during the current study are available from the corresponding author on reasonable request.

## Abbreviations

aOR: Adjusted odds ratio
CFPC: Couples’ family planning counseling
CHW: Community health worker
CVCT: Couples’ voluntary HIV counseling and testing
IUD: Intrauterine device
LARC: Long-acting reversible contraception
MoH: Ministry of Health
OCP: Oral contraceptive pills
PSF: Projet San Francisco
RZHRG: Rwanda Zambia HIV Research Group
